# Supplementary material for: How accurate are WorldPop-Global-Unconstrained gridded population data at the cell-level?: A simulation analysis in urban Namibia
Source: PLoS One. 2022 Jul 21;17(7):e0271504. doi: 10.1371/journal.pone.0271504 (PMC9302737; doi:10.1371/journal.pone.0271504)
Supplement: S1 Table — (DOCX) [file pone.0271504.s001.docx]

**S3 Table.** Percent of population missing from LMIC censuses by source

|  | Agarwal (2011) | Carr-Hill (2013) | Carr-Hill (2017) | Ebenstein (2015) | Gidado (2013) | Gurgel (2003) | Jiang (2015) | Karanja (2010) | Kronefeld (2008) | Lucci  (2018) | Sabry (2010) |
| --- | --- | --- | --- | --- | --- | --- | --- | --- | --- | --- | --- |
| Location & Housing Type | India | LMICs | Tanzania, Kenya, Uganda | China | Nigeria | Brazil  (kids only) | China | Kenya | Afghan-istan | Kenya | Egypt |
| Urban slum |  |  |  |  |  |  |  |  |  |  |  |
| Permanent/semi-permanent (not mobile) | 50% | 5-13% | 17-51% |  |  |  |  | 21% |  | 18, 21, 38, 59% | 45% |
| Permanent/semi-permanent (mobile) |  |  |  | 33-61% |  |  |  |  | 44% |  |  |
| Homeless |  | 100% |  | 33-61% |  | 64% |  |  |  |  |  |
| Urban non-slum |  |  |  |  |  |  |  |  |  |  |  |
| Permanent |  |  |  |  |  |  | 2% |  |  |  |  |
| Rural |  |  |  |  |  |  |  |  |  |  |  |
| Permanent/semi-permanent |  |  |  |  |  |  | 2% |  |  |  |  |
| Remote |  |  |  |  | 12% |  |  |  |  |  |  |
| Nomad |  |  |  |  | 12% |  |  |  |  |  |  |
| Institutional |  |  |  |  |  |  |  |  |  |  |  |
| Hospital / care home |  | <1% |  |  |  |  |  |  |  |  |  |
| Prison |  | 0.09 |  |  |  |  |  |  |  |  |  |
| Refugee camp |  | 10-15% |  |  |  |  |  |  |  |  |  |
| Citation | [55] | [7] | [59] | [60] | [61] | [62] | [63] | [64] | [65] | [66] | [56] |
| *continued…* | | | | | | | | | | | |

| *continued…* |  |  |  |  |  |  |  |  |  |  |  |  |  |
| --- | --- | --- | --- | --- | --- | --- | --- | --- | --- | --- | --- | --- | --- |
|  | | Stark  (2017) | Treiman (2005) | PES (2000) | PES (2001) | PES (2002) | PES (2002) | PES (2010) | PES (2010) | PES (2010) | PES  (2011) | PES (2011) | PES (2011) |
| Location & Housing Type | | Cambodia  (kids only) | China | Brazil | Nepal | Tanzania | Uganda | Ghana | Zambia | Rwanda | Bangladesh | India | South Africa |
| Urban slum | |  |  |  |  |  |  |  |  |  |  |  |  |
| Permanent/semi-permanent (not mobile) | |  |  |  |  |  |  |  |  |  |  |  |  |
| Permanent/semi-permanent (mobile) | |  | 50% |  |  |  |  |  |  |  |  |  |  |
| Homeless | | 80-96% | 50% |  |  |  |  |  |  |  |  |  |  |
| Urban non-slum | |  |  |  |  |  |  |  |  |  |  |  |  |
| Permanent | |  |  | 2-4% | 12% | 7% | 12% | 3% | 5% | 2% | 5% | 3% | 15% |
| Rural | |  |  |  |  |  |  |  |  |  |  |  |  |
| Permanent/semi-permanent | |  |  | 4-11% | 5% | 7% | 5% | 2% | 10% | 2% | 4% | 2% | 13% |
| Remote | |  |  |  |  |  |  |  |  |  |  |  |  |
| Nomad | |  |  |  |  |  |  |  |  |  |  |  |  |
| Institutional | |  |  |  |  |  |  |  |  |  |  |  |  |
| Hospital / care home | |  |  |  |  |  |  |  |  |  |  |  |  |
| Prison | |  |  |  |  |  |  |  |  |  |  |  |  |
| Refugee camp | |  |  |  |  |  |  |  |  |  |  |  |  |
| Citation | | [57] | [58] | [45] | [46] | [47] | [48] | [49] | [50] | [54] | [51] | [52] | [53] |
