# Supplementary material for: How accurate are WorldPop-Global-Unconstrained gridded population data at the cell-level?: A simulation analysis in urban Namibia
Source: PLoS One. 2022 Jul 21;17(7):e0271504. doi: 10.1371/journal.pone.0271504 (PMC9302737; doi:10.1371/journal.pone.0271504)
Supplement: S2 Table — (DOCX) [file pone.0271504.s002.docx]

**S5 Table**. Root Mean Square Error (RMSE) statistics for all scenarios

| **Area-Adjusted RMSE**, EA-level input data, excluding cells with estimated population <1 | | | | | | | | | | | | | | | | |
| --- | --- | --- | --- | --- | --- | --- | --- | --- | --- | --- | --- | --- | --- | --- | --- | --- |
| Cell size | **2016_True** | **2016_L** | **2016_M** | **2016_H** | **2011_True** | **2011_L** | **2011_M** | **2011_H** | **2006_True** | **2006_L** | **2006_M** | **2006_H** | **2001_True** | **2001_L** | **2001_M** | **2001_H** |
| (metres) | **Non-slum** |  |  |  |  |  |  |  |  |  |  |  |  |  |  |  |
| 100 | 21.4 | 21.3 | 21.3 | 21.7 | 21.6 | 21.6 | 21.7 | 22.3 | 22.4 | 22.5 | 22.9 | 24.1 | 23.8 | 24.1 | 24.7 | 26.2 |
| 200 | 31.8 | 32.0 | 32.4 | 33.1 | 32.2 | 32.4 | 32.8 | 33.5 | 33.2 | 33.5 | 34.0 | 34.9 | 35.0 | 35.3 | 35.8 | 36.4 |
| 300 | 35.0 | 35.2 | 35.5 | 35.9 | 35.3 | 35.4 | 35.7 | 36.2 | 36.1 | 36.3 | 36.5 | 37.1 | 37.6 | 37.7 | 38.0 | 38.4 |
| 400 | 35.1 | 35.3 | 35.4 | 35.6 | 35.4 | 35.4 | 35.5 | 35.8 | 36.2 | 36.3 | 36.4 | 36.8 | 37.2 | 37.3 | 37.6 | 37.8 |
| 500 | 35.0 | 35.1 | 35.2 | 35.5 | 35.1 | 35.2 | 35.3 | 35.6 | 35.8 | 35.8 | 36.1 | 36.2 | 36.7 | 36.9 | 37.1 | 37.2 |
| 600 | 33.8 | 33.9 | 33.9 | 34.1 | 33.9 | 34.0 | 33.9 | 34.1 | 34.1 | 34.1 | 34.3 | 34.6 | 35.1 | 35.2 | 35.4 | 35.5 |
| 700 | 35.1 | 35.1 | 35.2 | 35.4 | 35.4 | 35.3 | 35.4 | 35.5 | 35.7 | 35.7 | 35.8 | 35.9 | 36.8 | 36.9 | 36.9 | 37.0 |
| 800 | 35.1 | 35.1 | 35.1 | 35.5 | 35.1 | 35.1 | 35.3 | 35.4 | 35.6 | 35.6 | 35.8 | 35.9 | 36.7 | 36.6 | 36.9 | 37.0 |
| 900 | 33.8 | 33.8 | 33.8 | 34.0 | 33.9 | 33.9 | 34.0 | 34.1 | 34.1 | 34.1 | 34.3 | 34.5 | 35.2 | 35.2 | 35.3 | 35.4 |
| 1000 | 29.9 | 29.9 | 29.9 | 30.1 | 29.9 | 29.9 | 29.9 | 30.1 | 30.2 | 30.2 | 30.2 | 30.2 | 31.0 | 31.0 | 31.0 | 31.0 |
|  | **Slum** |  |  |  |  |  |  |  |  |  |  |  |  |  |  |  |
| 100 | 32.8 | 33.0 | 34.4 | 46.6 | 32.1 | 33.0 | 37.9 | 52.9 | 39.9 | 41.2 | 47.5 | 64.7 | 43.0 | 45.3 | 53.7 | 72.2 |
| 200 | 47.0 | 48.6 | 51.9 | 58.2 | 52.5 | 54.1 | 57.4 | 63.8 | 60.7 | 62.6 | 67.5 | 75.9 | 64.9 | 66.8 | 72.6 | 82.5 |
| 300 | 52.2 | 52.9 | 54.6 | 58.5 | 57.3 | 58.1 | 59.6 | 63.8 | 64.3 | 65.5 | 69.3 | 76.8 | 66.6 | 67.3 | 73.3 | 81.2 |
| 400 | 55.4 | 55.8 | 57.0 | 59.8 | 59.8 | 60.3 | 61.4 | 65.2 | 67.1 | 68.2 | 70.8 | 77.1 | 72.1 | 72.8 | 76.6 | 84.7 |
| 500 | 53.8 | 54.5 | 55.5 | 57.8 | 58.3 | 58.5 | 59.6 | 61.9 | 64.3 | 65.4 | 69.4 | 75.6 | 67.8 | 67.9 | 74.6 | 79.4 |
| 600 | 54.4 | 54.6 | 55.2 | 58.6 | 57.3 | 58.6 | 59.3 | 62.5 | 67.3 | 69.0 | 70.1 | 74.2 | 69.7 | 69.8 | 73.9 | 78.5 |
| 700 | 54.8 | 54.9 | 55.1 | 57.8 | 58.3 | 59.1 | 59.3 | 61.0 | 64.9 | 66.2 | 67.6 | 71.9 | 68.3 | 68.4 | 71.5 | 75.6 |
| 800 | 52.9 | 53.0 | 53.1 | 54.8 | 55.9 | 56.0 | 57.4 | 58.1 | 62.3 | 66.5 | 68.2 | 72.6 | 65.0 | 66.6 | 71.4 | 80.3 |
| 900 | 50.1 | 50.2 | 51.1 | 54.0 | 52.8 | 52.8 | 52.9 | 56.2 | 61.4 | 63.2 | 65.2 | 67.5 | 62.5 | 62.5 | 66.6 | 71.6 |
| 1000 | 56.1 | 56.2 | 57.3 | 58.5 | 59.4 | 59.4 | 60.4 | 63.2 | 67.8 | 69.9 | 72.1 | 74.8 | 74.0 | 74.1 | 74.1 | 80.1 |
|  | **Rural** |  |  |  |  |  |  |  |  |  |  |  |  |  |  |  |
| 100 | 29.2 | 32.9 | 26.7 | 29.9 | 31.0 | 33.7 | 38.3 | 30.5 | 34.9 | 34.0 | 29.9 | 31.0 | 33.2 | 31.7 | 30.2 | 30.6 |
| 200 | 7.4 | 8.3 | 6.9 | 7.6 | 7.9 | 8.5 | 9.6 | 7.8 | 8.8 | 8.5 | 7.6 | 7.9 | 8.3 | 7.9 | 7.6 | 7.7 |
| 300 | 4.0 | 4.3 | 3.8 | 4.1 | 4.2 | 4.4 | 4.8 | 4.2 | 4.4 | 4.3 | 3.9 | 4.1 | 4.2 | 4.0 | 3.9 | 4.0 |
| 400 | 3.2 | 3.3 | 3.1 | 3.2 | 3.3 | 3.4 | 3.5 | 3.3 | 3.3 | 3.3 | 3.2 | 3.2 | 3.2 | 3.1 | 3.1 | 3.1 |
| 500 | 2.8 | 2.9 | 2.8 | 2.9 | 2.8 | 2.9 | 2.9 | 2.9 | 2.5 | 2.5 | 2.5 | 2.5 | 2.3 | 2.3 | 2.2 | 2.2 |
| 600 | 4.2 | 4.3 | 4.3 | 4.3 | 4.2 | 4.3 | 4.3 | 4.3 | 3.8 | 3.8 | 3.8 | 3.9 | 3.6 | 3.6 | 3.6 | 3.7 |
| 700 | 3.6 | 3.7 | 3.7 | 3.7 | 3.6 | 3.6 | 3.6 | 3.6 | 3.3 | 3.3 | 3.3 | 3.4 | 3.0 | 3.0 | 3.0 | 3.0 |
| 800 | 4.3 | 4.4 | 4.4 | 4.4 | 4.4 | 4.4 | 4.4 | 4.4 | 4.1 | 4.1 | 4.1 | 4.2 | 4.1 | 4.1 | 4.1 | 4.1 |
| 900 | 5.2 | 5.2 | 5.2 | 5.3 | 5.2 | 5.2 | 5.3 | 5.3 | 4.5 | 4.5 | 4.5 | 4.6 | 3.8 | 3.8 | 3.8 | 3.8 |
| 1000 | 5.3 | 5.3 | 5.4 | 5.4 | 5.4 | 5.4 | 5.4 | 5.4 | 5.4 | 5.4 | 5.4 | 5.4 | 5.4 | 5.4 | 5.4 | 5.4 |

|  | **Area-Adjusted RMSE**, Constituency-level input data, excluding cells with estimated population <1 | | | | | | | | | | | | | | | | |
| --- | --- | --- | --- | --- | --- | --- | --- | --- | --- | --- | --- | --- | --- | --- | --- | --- | --- |
| Cell size | | **2016_True** | **2016_L** | **2016_M** | **2016_H** | **2011_True** | **2011_L** | **2011_M** | **2011_H** | **2006_True** | **2006_L** | **2006_M** | **2006_H** | **2001_True** | **2001_L** | **2001_M** | **2001_H** |
| (metres) | | **Non-slum** |  |  |  |  |  |  |  |  |  |  |  |  |  |  |  |
| 100 | | 28.0 | 27.9 | 28.9 | 29.8 | 28.3 | 28.7 | 29.1 | 30.1 | 29.9 | 30.1 | 30.6 | 31.8 | 31.8 | 32.0 | 32.4 | 32.9 |
| 200 | | 32.8 | 32.9 | 33.5 | 34.0 | 33.1 | 33.4 | 33.7 | 34.2 | 34.0 | 34.2 | 34.5 | 35.1 | 35.2 | 35.3 | 35.5 | 35.7 |
| 300 | | 34.0 | 34.0 | 34.4 | 34.7 | 34.2 | 34.4 | 34.5 | 34.8 | 34.7 | 34.8 | 35.0 | 35.4 | 35.5 | 35.6 | 35.6 | 35.8 |
| 400 | | 33.4 | 33.4 | 33.8 | 34.0 | 33.7 | 33.8 | 33.9 | 34.1 | 34.0 | 34.1 | 34.3 | 34.6 | 34.6 | 34.6 | 34.7 | 34.8 |
| 500 | | 33.1 | 33.1 | 33.5 | 33.6 | 33.3 | 33.5 | 33.6 | 33.6 | 33.6 | 33.7 | 34.0 | 34.1 | 34.2 | 34.2 | 34.2 | 34.3 |
| 600 | | 31.8 | 31.8 | 32.0 | 32.2 | 31.9 | 32.0 | 32.1 | 32.3 | 32.3 | 32.3 | 32.4 | 32.6 | 32.6 | 32.7 | 32.6 | 32.7 |
| 700 | | 33.4 | 33.4 | 33.7 | 33.7 | 33.6 | 33.7 | 33.7 | 33.8 | 33.9 | 33.9 | 34.1 | 34.1 | 34.2 | 34.2 | 34.2 | 34.3 |
| 800 | | 33.1 | 33.1 | 33.3 | 33.4 | 33.3 | 33.3 | 33.4 | 33.4 | 33.4 | 33.6 | 33.8 | 33.8 | 33.8 | 33.9 | 33.9 | 33.9 |
| 900 | | 32.4 | 32.3 | 32.4 | 32.5 | 32.4 | 32.4 | 32.4 | 32.5 | 32.5 | 32.5 | 32.5 | 32.7 | 32.7 | 32.7 | 32.7 | 32.7 |
| 1000 | | 28.7 | 28.7 | 28.7 | 28.7 | 28.7 | 28.7 | 28.7 | 28.7 | 28.7 | 28.8 | 29.0 | 29.0 | 29.0 | 29.0 | 29.0 | 29.0 |
|  | | **Slum** |  |  |  |  |  |  |  |  |  |  |  |  |  |  |  |
| 100 | | 54.3 | 55.1 | 57.9 | 62.4 | 56.1 | 57.4 | 59.6 | 63.3 | 61.0 | 61.6 | 63.5 | 66.8 | 63.6 | 64.6 | 65.9 | 67.5 |
| 200 | | 65.5 | 65.7 | 66.6 | 67.9 | 66.0 | 66.4 | 67.1 | 68.2 | 67.5 | 67.7 | 68.2 | 69.1 | 68.3 | 68.5 | 68.9 | 69.5 |
| 300 | | 65.4 | 65.5 | 65.9 | 66.5 | 65.7 | 65.8 | 66.2 | 66.6 | 66.3 | 66.4 | 66.7 | 67.1 | 66.7 | 66.8 | 67.0 | 67.3 |
| 400 | | 67.2 | 67.3 | 67.5 | 67.8 | 67.3 | 67.4 | 67.6 | 67.9 | 67.7 | 67.8 | 67.9 | 68.1 | 67.9 | 68.0 | 68.1 | 68.2 |
| 500 | | 64.6 | 64.6 | 64.8 | 65.0 | 64.7 | 64.7 | 64.9 | 65.0 | 64.9 | 64.9 | 65.0 | 65.2 | 65.0 | 65.1 | 65.1 | 65.2 |
| 600 | | 66.0 | 66.1 | 66.2 | 66.3 | 66.1 | 66.2 | 66.2 | 66.4 | 66.3 | 66.3 | 66.4 | 66.5 | 66.4 | 66.4 | 66.5 | 66.5 |
| 700 | | 63.7 | 63.7 | 63.8 | 63.9 | 63.7 | 63.8 | 63.8 | 63.9 | 63.9 | 63.9 | 63.9 | 64.0 | 63.9 | 63.9 | 64.0 | 64.0 |
| 800 | | 60.1 | 60.1 | 60.1 | 60.2 | 60.1 | 60.1 | 60.2 | 60.2 | 60.2 | 60.2 | 60.2 | 60.3 | 60.3 | 60.3 | 60.3 | 61.3 |
| 900 | | 59.8 | 59.8 | 59.9 | 60.0 | 59.9 | 59.9 | 59.9 | 60.0 | 59.9 | 60.0 | 60.0 | 60.0 | 60.0 | 60.0 | 60.0 | 60.0 |
| 1000 | | 65.1 | 65.1 | 65.2 | 65.2 | 65.1 | 65.2 | 65.2 | 65.2 | 65.2 | 65.2 | 65.2 | 65.3 | 65.2 | 65.2 | 65.3 | 65.3 |
|  | | **Rural** |  |  |  |  |  |  |  |  |  |  |  |  |  |  |  |
| 100 | | 10.7 | 9.8 | 8.6 | 6.7 | 9.3 | 9.0 | 7.7 | 6.2 | 7.2 | 6.8 | 5.9 | 5.1 | 6.2 | 5.8 | 5.2 | 4.5 |
| 200 | | 3.2 | 3.1 | 2.9 | 2.7 | 3.0 | 3.0 | 2.8 | 2.7 | 2.8 | 2.8 | 2.7 | 2.7 | 2.8 | 2.8 | 2.8 | 2.9 |
| 300 | | 2.7 | 2.8 | 2.7 | 2.8 | 2.7 | 2.8 | 2.8 | 2.9 | 2.8 | 2.9 | 2.9 | 3.0 | 3.0 | 3.0 | 3.1 | 3.3 |
| 400 | | 2.7 | 2.8 | 2.8 | 2.9 | 2.7 | 2.8 | 2.8 | 2.9 | 2.9 | 2.9 | 2.9 | 3.1 | 3.1 | 3.0 | 3.1 | 3.3 |
| 500 | | 2.7 | 2.7 | 2.7 | 2.8 | 2.7 | 2.7 | 2.8 | 2.9 | 2.8 | 2.8 | 2.8 | 3.0 | 3.0 | 3.0 | 3.1 | 3.2 |
| 600 | | 4.4 | 4.5 | 4.5 | 4.6 | 4.4 | 4.5 | 4.6 | 4.7 | 4.6 | 4.6 | 4.6 | 4.9 | 4.9 | 4.8 | 5.0 | 5.3 |
| 700 | | 4.0 | 4.0 | 4.0 | 4.1 | 4.0 | 4.1 | 4.1 | 4.2 | 4.1 | 4.2 | 4.1 | 4.3 | 4.4 | 4.3 | 4.4 | 4.7 |
| 800 | | 4.6 | 4.7 | 4.7 | 4.9 | 4.7 | 4.8 | 4.9 | 5.0 | 4.9 | 5.0 | 5.0 | 5.2 | 5.2 | 5.2 | 5.3 | 5.6 |
| 900 | | 5.6 | 5.7 | 5.8 | 6.0 | 5.7 | 5.7 | 5.9 | 6.1 | 5.9 | 6.0 | 6.1 | 6.5 | 6.4 | 6.3 | 6.4 | 6.8 |
| 1000 | | 5.9 | 6.0 | 6.0 | 6.2 | 5.9 | 6.0 | 6.2 | 6.4 | 6.2 | 6.2 | 6.3 | 6.7 | 6.6 | 6.6 | 6.8 | 7.2 |

| **Population-Adjusted RMSE**, EA-level input data, excluding cells with estimated population <1 | | | | | | | | | | | | | | | | |
| --- | --- | --- | --- | --- | --- | --- | --- | --- | --- | --- | --- | --- | --- | --- | --- | --- |
| Cell size | **2016_True** | **2016_L** | **2016_M** | **2016_H** | **2011_True** | **2011_L** | **2011_M** | **2011_H** | **2006_True** | **2006_L** | **2006_M** | **2006_H** | **2001_True** | **2001_L** | **2001_M** | **2001_H** |
| (metres) | **Non-slum** |  |  |  |  |  |  |  |  |  |  |  |  |  |  |  |
| 100 | 0.549 | 0.548 | 0.548 | 0.557 | 0.556 | 0.556 | 0.557 | 0.572 | 0.576 | 0.578 | 0.589 | 0.619 | 0.612 | 0.618 | 0.634 | 0.673 |
| 200 | 1.031 | 1.040 | 1.053 | 1.074 | 1.044 | 1.052 | 1.065 | 1.089 | 1.079 | 1.089 | 1.103 | 1.134 | 1.138 | 1.146 | 1.162 | 1.182 |
| 300 | 1.272 | 1.281 | 1.290 | 1.307 | 1.283 | 1.287 | 1.298 | 1.316 | 1.315 | 1.320 | 1.328 | 1.350 | 1.366 | 1.370 | 1.383 | 1.398 |
| 400 | 1.380 | 1.386 | 1.389 | 1.400 | 1.390 | 1.389 | 1.395 | 1.408 | 1.422 | 1.425 | 1.429 | 1.446 | 1.462 | 1.465 | 1.477 | 1.484 |
| 500 | 1.461 | 1.466 | 1.471 | 1.482 | 1.466 | 1.470 | 1.476 | 1.488 | 1.497 | 1.498 | 1.507 | 1.512 | 1.535 | 1.541 | 1.550 | 1.556 |
| 600 | 1.519 | 1.523 | 1.521 | 1.530 | 1.522 | 1.526 | 1.525 | 1.531 | 1.533 | 1.534 | 1.539 | 1.554 | 1.579 | 1.580 | 1.588 | 1.594 |
| 700 | 1.561 | 1.562 | 1.564 | 1.574 | 1.574 | 1.571 | 1.572 | 1.579 | 1.588 | 1.589 | 1.591 | 1.597 | 1.635 | 1.640 | 1.641 | 1.643 |
| 800 | 1.635 | 1.636 | 1.637 | 1.655 | 1.636 | 1.636 | 1.643 | 1.650 | 1.659 | 1.660 | 1.667 | 1.675 | 1.709 | 1.704 | 1.717 | 1.726 |
| 900 | 1.576 | 1.576 | 1.577 | 1.585 | 1.583 | 1.583 | 1.584 | 1.592 | 1.591 | 1.591 | 1.599 | 1.607 | 1.643 | 1.644 | 1.644 | 1.653 |
| 1000 | 1.622 | 1.622 | 1.623 | 1.632 | 1.622 | 1.623 | 1.623 | 1.632 | 1.639 | 1.640 | 1.640 | 1.641 | 1.681 | 1.681 | 1.681 | 1.682 |
|  | **Slum** |  |  |  |  |  |  |  |  |  |  |  |  |  |  |  |
| 100 | 0.608 | 0.612 | 0.638 | 0.863 | 0.595 | 0.611 | 0.702 | 0.981 | 0.740 | 0.764 | 0.881 | 1.199 | 0.797 | 0.839 | 0.996 | 1.338 |
| 200 | 1.030 | 1.065 | 1.138 | 1.277 | 1.151 | 1.186 | 1.259 | 1.401 | 1.331 | 1.373 | 1.480 | 1.666 | 1.424 | 1.466 | 1.593 | 1.810 |
| 300 | 1.246 | 1.263 | 1.303 | 1.397 | 1.368 | 1.388 | 1.424 | 1.525 | 1.536 | 1.564 | 1.657 | 1.834 | 1.592 | 1.607 | 1.751 | 1.940 |
| 400 | 1.366 | 1.374 | 1.405 | 1.472 | 1.473 | 1.486 | 1.513 | 1.607 | 1.654 | 1.680 | 1.745 | 1.899 | 1.777 | 1.795 | 1.887 | 2.086 |
| 500 | 1.403 | 1.422 | 1.447 | 1.508 | 1.521 | 1.526 | 1.554 | 1.615 | 1.676 | 1.706 | 1.809 | 1.972 | 1.768 | 1.771 | 1.947 | 2.072 |
| 600 | 1.442 | 1.445 | 1.462 | 1.553 | 1.517 | 1.553 | 1.571 | 1.656 | 1.782 | 1.828 | 1.857 | 1.966 | 1.846 | 1.849 | 1.959 | 2.079 |
| 700 | 1.465 | 1.468 | 1.473 | 1.546 | 1.559 | 1.580 | 1.586 | 1.633 | 1.737 | 1.771 | 1.810 | 1.923 | 1.827 | 1.829 | 1.913 | 2.023 |
| 800 | 1.523 | 1.525 | 1.529 | 1.577 | 1.610 | 1.612 | 1.651 | 1.672 | 1.794 | 1.914 | 1.962 | 2.090 | 1.870 | 1.917 | 2.055 | 2.311 |
| 900 | 1.441 | 1.442 | 1.470 | 1.554 | 1.518 | 1.519 | 1.522 | 1.616 | 1.764 | 1.817 | 1.875 | 1.940 | 1.797 | 1.798 | 1.916 | 2.059 |
| 1000 | 1.375 | 1.377 | 1.404 | 1.434 | 1.456 | 1.457 | 1.481 | 1.550 | 1.663 | 1.715 | 1.768 | 1.833 | 1.815 | 1.816 | 1.818 | 1.964 |
|  | **Rural** |  |  |  |  |  |  |  |  |  |  |  |  |  |  |  |
| 100 | 5.044 | 5.680 | 4.599 | 5.155 | 5.352 | 5.818 | 6.610 | 5.257 | 6.024 | 5.858 | 5.155 | 5.349 | 5.727 | 5.462 | 5.203 | 5.273 |
| 200 | 3.208 | 3.591 | 2.960 | 3.293 | 3.394 | 3.671 | 4.151 | 3.363 | 3.783 | 3.688 | 3.272 | 3.401 | 3.582 | 3.422 | 3.271 | 3.311 |
| 300 | 2.794 | 3.025 | 2.684 | 2.882 | 2.924 | 3.095 | 3.386 | 2.939 | 3.067 | 3.000 | 2.752 | 2.846 | 2.917 | 2.822 | 2.737 | 2.774 |
| 400 | 3.110 | 3.236 | 3.071 | 3.201 | 3.202 | 3.306 | 3.476 | 3.250 | 3.289 | 3.266 | 3.152 | 3.201 | 3.154 | 3.090 | 3.051 | 3.090 |
| 500 | 3.699 | 3.817 | 3.694 | 3.801 | 3.737 | 3.793 | 3.904 | 3.785 | 3.380 | 3.366 | 3.284 | 3.253 | 3.043 | 3.005 | 2.978 | 2.900 |
| 600 | 5.386 | 5.432 | 5.415 | 5.511 | 5.379 | 5.417 | 5.463 | 5.458 | 4.881 | 4.888 | 4.870 | 4.926 | 4.614 | 4.601 | 4.619 | 4.667 |
| 700 | 5.521 | 5.550 | 5.572 | 5.631 | 5.415 | 5.430 | 5.488 | 5.508 | 5.049 | 5.054 | 5.061 | 5.106 | 4.543 | 4.550 | 4.530 | 4.537 |
| 800 | 6.549 | 6.566 | 6.579 | 6.664 | 6.602 | 6.649 | 6.676 | 6.696 | 6.231 | 6.246 | 6.256 | 6.268 | 6.124 | 6.135 | 6.142 | 6.173 |
| 900 | 8.030 | 8.075 | 8.137 | 8.195 | 8.133 | 8.109 | 8.158 | 8.260 | 7.014 | 7.050 | 7.058 | 7.085 | 5.845 | 5.843 | 5.849 | 5.854 |
| 1000 | 10.220 | 10.255 | 10.429 | 10.554 | 10.420 | 10.453 | 10.556 | 10.594 | 10.536 | 10.539 | 10.544 | 10.558 | 10.432 | 10.435 | 10.441 | 10.485 |

|  | **Population-Adjusted RMSE**, Constituency-level input data, excluding cells with estimated population <1 | | | | | | | | | | | | | | | | |
| --- | --- | --- | --- | --- | --- | --- | --- | --- | --- | --- | --- | --- | --- | --- | --- | --- | --- |
| Cell size | | **2016_True** | **2016_L** | **2016_M** | **2016_H** | **2011_True** | **2011_L** | **2011_M** | **2011_H** | **2006_True** | **2006_L** | **2006_M** | **2006_H** | **2001_True** | **2001_L** | **2001_M** | **2001_H** |
| (metres) | | **Non-Slum** |  |  |  |  |  |  |  |  |  |  |  |  |  |  |  |
| 100 | | 0.719 | 0.717 | 0.743 | 0.766 | 0.727 | 0.738 | 0.748 | 0.774 | 0.769 | 0.773 | 0.787 | 0.816 | 0.816 | 0.822 | 0.831 | 0.844 |
| 200 | | 1.065 | 1.066 | 1.087 | 1.105 | 1.075 | 1.084 | 1.092 | 1.111 | 1.105 | 1.109 | 1.118 | 1.139 | 1.143 | 1.147 | 1.152 | 1.159 |
| 300 | | 1.235 | 1.235 | 1.252 | 1.262 | 1.244 | 1.250 | 1.255 | 1.266 | 1.262 | 1.265 | 1.274 | 1.288 | 1.291 | 1.294 | 1.296 | 1.301 |
| 400 | | 1.313 | 1.311 | 1.328 | 1.336 | 1.323 | 1.328 | 1.331 | 1.338 | 1.336 | 1.340 | 1.346 | 1.358 | 1.359 | 1.360 | 1.362 | 1.366 |
| 500 | | 1.385 | 1.386 | 1.400 | 1.404 | 1.391 | 1.398 | 1.403 | 1.405 | 1.403 | 1.409 | 1.419 | 1.427 | 1.428 | 1.428 | 1.430 | 1.433 |
| 600 | | 1.430 | 1.430 | 1.439 | 1.449 | 1.433 | 1.439 | 1.442 | 1.452 | 1.451 | 1.453 | 1.454 | 1.463 | 1.464 | 1.467 | 1.466 | 1.469 |
| 700 | | 1.485 | 1.483 | 1.497 | 1.499 | 1.493 | 1.497 | 1.498 | 1.502 | 1.505 | 1.509 | 1.517 | 1.515 | 1.518 | 1.519 | 1.519 | 1.524 |
| 800 | | 1.542 | 1.542 | 1.552 | 1.557 | 1.551 | 1.552 | 1.556 | 1.558 | 1.557 | 1.566 | 1.576 | 1.577 | 1.577 | 1.578 | 1.578 | 1.579 |
| 900 | | 1.510 | 1.505 | 1.511 | 1.517 | 1.510 | 1.511 | 1.511 | 1.517 | 1.517 | 1.517 | 1.518 | 1.524 | 1.524 | 1.525 | 1.525 | 1.526 |
| 1000 | | 1.557 | 1.557 | 1.558 | 1.559 | 1.557 | 1.558 | 1.558 | 1.559 | 1.559 | 1.564 | 1.572 | 1.573 | 1.573 | 1.573 | 1.574 | 1.574 |
|  | | **Slum** |  |  |  |  |  |  |  |  |  |  |  |  |  |  |  |
| 100 | | 1.006 | 1.022 | 1.074 | 1.157 | 1.039 | 1.063 | 1.105 | 1.174 | 1.131 | 1.142 | 1.177 | 1.238 | 1.179 | 1.196 | 1.221 | 1.251 |
| 200 | | 1.436 | 1.442 | 1.461 | 1.490 | 1.449 | 1.457 | 1.472 | 1.496 | 1.481 | 1.485 | 1.496 | 1.517 | 1.497 | 1.503 | 1.511 | 1.524 |
| 300 | | 1.562 | 1.565 | 1.575 | 1.589 | 1.569 | 1.573 | 1.580 | 1.592 | 1.585 | 1.586 | 1.592 | 1.602 | 1.592 | 1.595 | 1.599 | 1.608 |
| 400 | | 1.655 | 1.657 | 1.662 | 1.671 | 1.659 | 1.661 | 1.666 | 1.673 | 1.668 | 1.669 | 1.673 | 1.679 | 1.673 | 1.675 | 1.677 | 1.680 |
| 500 | | 1.684 | 1.685 | 1.689 | 1.695 | 1.687 | 1.688 | 1.691 | 1.696 | 1.693 | 1.694 | 1.696 | 1.700 | 1.696 | 1.697 | 1.699 | 1.701 |
| 600 | | 1.749 | 1.750 | 1.753 | 1.757 | 1.751 | 1.753 | 1.755 | 1.758 | 1.756 | 1.757 | 1.759 | 1.761 | 1.759 | 1.760 | 1.761 | 1.762 |
| 700 | | 1.704 | 1.704 | 1.706 | 1.709 | 1.705 | 1.706 | 1.707 | 1.710 | 1.708 | 1.709 | 1.710 | 1.712 | 1.710 | 1.710 | 1.711 | 1.713 |
| 800 | | 1.728 | 1.729 | 1.731 | 1.733 | 1.730 | 1.730 | 1.732 | 1.734 | 1.733 | 1.733 | 1.734 | 1.735 | 1.734 | 1.735 | 1.735 | 1.766 |
| 900 | | 1.720 | 1.721 | 1.722 | 1.724 | 1.721 | 1.722 | 1.723 | 1.725 | 1.724 | 1.724 | 1.725 | 1.726 | 1.725 | 1.725 | 1.726 | 1.726 |
| 1000 | | 1.596 | 1.596 | 1.597 | 1.599 | 1.597 | 1.597 | 1.598 | 1.599 | 1.598 | 1.599 | 1.599 | 1.600 | 1.599 | 1.600 | 1.600 | 1.601 |
|  | | **Rural** |  |  |  |  |  |  |  |  |  |  |  |  |  |  |  |
| 100 | | 1.839 | 1.698 | 1.484 | 1.153 | 1.608 | 1.549 | 1.329 | 1.073 | 1.248 | 1.168 | 1.013 | 0.878 | 1.077 | 0.994 | 0.901 | 0.770 |
| 200 | | 1.402 | 1.353 | 1.265 | 1.176 | 1.309 | 1.295 | 1.224 | 1.177 | 1.211 | 1.194 | 1.150 | 1.180 | 1.218 | 1.190 | 1.191 | 1.234 |
| 300 | | 1.913 | 1.932 | 1.922 | 1.975 | 1.915 | 1.934 | 1.963 | 2.027 | 1.989 | 2.005 | 2.001 | 2.120 | 2.118 | 2.088 | 2.149 | 2.290 |
| 400 | | 2.682 | 2.734 | 2.727 | 2.817 | 2.708 | 2.744 | 2.788 | 2.887 | 2.821 | 2.849 | 2.853 | 3.038 | 3.006 | 2.981 | 3.061 | 3.266 |
| 500 | | 3.527 | 3.590 | 3.601 | 3.710 | 3.566 | 3.612 | 3.675 | 3.802 | 3.708 | 3.742 | 3.756 | 3.999 | 3.956 | 3.938 | 4.051 | 4.303 |
| 600 | | 5.597 | 5.702 | 5.692 | 5.872 | 5.664 | 5.697 | 5.795 | 6.014 | 5.830 | 5.899 | 5.910 | 6.274 | 6.228 | 6.149 | 6.320 | 6.802 |
| 700 | | 5.995 | 6.112 | 6.113 | 6.260 | 6.074 | 6.164 | 6.213 | 6.385 | 6.257 | 6.284 | 6.283 | 6.567 | 6.613 | 6.578 | 6.693 | 7.163 |
| 800 | | 7.015 | 7.154 | 7.114 | 7.424 | 7.078 | 7.190 | 7.340 | 7.598 | 7.381 | 7.495 | 7.517 | 7.895 | 7.861 | 7.802 | 7.938 | 8.485 |
| 900 | | 8.684 | 8.913 | 8.950 | 9.294 | 8.843 | 8.920 | 9.144 | 9.477 | 9.206 | 9.382 | 9.421 | 10.017 | 9.869 | 9.805 | 9.913 | 10.610 |
| 1000 | | 11.480 | 11.769 | 11.615 | 12.063 | 11.563 | 11.737 | 12.003 | 12.393 | 12.064 | 12.159 | 12.206 | 13.044 | 12.817 | 12.808 | 13.158 | 13.928 |
