# Supplementary material for: How accurate are WorldPop-Global-Unconstrained gridded population data at the cell-level?: A simulation analysis in urban Namibia
Source: PLoS One. 2022 Jul 21;17(7):e0271504. doi: 10.1371/journal.pone.0271504 (PMC9302737; doi:10.1371/journal.pone.0271504)
Supplement: S1 File — (PDF) [file pone.0271504.s003.pdf]

Supplement 1. Simulating population in Khomas, Namibia

Supplement to Thomson DR, Leasure DR, Bird T, Tzavidis N, Tatem AJ. 2021. How accurate are WorldPop-Global-Unconstrained gridded population data at the cell-level?: A simulation analysis in urban Namibia.

The simulation in Khomas, Namibia followed the same steps outlined by Thomson and colleagues (2018)[1] for a simulated population in Oshikoto, Namibia:

(1) Use of a supervised clustering k-means algorithm to define realistic and distinct types of households in Khomas, Namibia based on eight variables in the 2013 Demographic and Health Survey (DHS) (Table S1.1, A) that were also present in a 20% census microdata sample (Table S1.1, B): urban, improved toilet, improved water source, sufficient sleeping space, durable structure, non-solid fuel for cooking, whether the head of household had any formal education, and whether there were any children under age five. A dendrogram showing the Euclidean distance between each pair of child clusters and their parent cluster in the k-means analysis indicated a sensible cut-off value of 1.0 to define four easy-to-interpret household types: urban poor, urban non-poor, rural poor, rural non-poor (Figure S1.1).

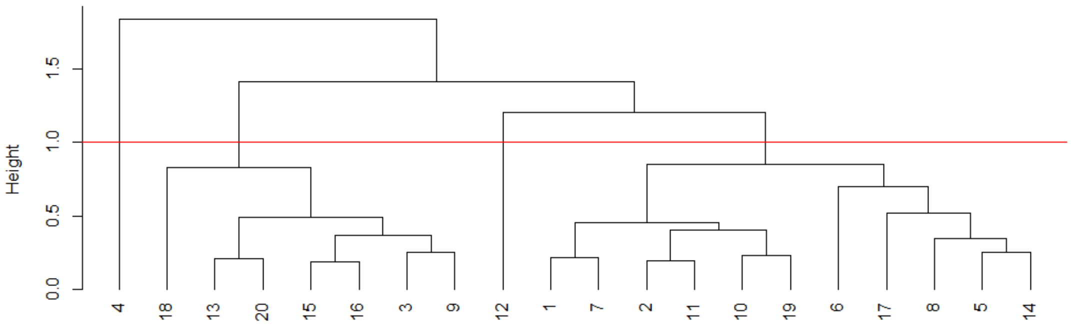

Cluster	rural	noedu	any_u5	unimp. toilet	unimp. water	inadeq. structure	limited space	solid fuel	Household type label
Type 1	0.00	0.02	0.30	0.30	0.01	0.08	0.10	0.01	Urban non-poor
Type 2	0.00	0.10	0.35	0.96	0.01	0.54	0.09	0.24	Urban poor
Type 3	1.00	0.33	0.40	1.00	0.13	0.53	0.14	1.00	Rural poor
Type 4	1.00	0.04	0.21	0.43	0.00	0.08	0.00	0.00	Rural non-poor
**Khomas**	0.06	0.05	0.31	0.50	0.01	0.21	0.09	0.09	

Figure S1.1. Dendrogram & k-mean scores of unique household types in Khomas, Namibia based on 2013 DHS

(2) Steps 2 and 3 involve prediction of household type probability surfaces. Although we only care about the household type probabilities in Khomas, we model probability surfaces for all of Namibia due to the limited number of 2013 DHS primary sampling units (PSUs) in Khomas (53 PSUs Khomas, 550 PSUs Namibia) available to train a model. Thus, in step 2, we processed 19 spatial auxiliary datasets available from free, public sources into 100x100m raster cells across all of Namibia, then calculated the average value within a 2km buffer from each cell (2km because the DHS randomly geo-displaces urban cluster coordinates by up to 2km) (Table S1.1).

[1] Thomson DR, Kools L, Jochem WC. 2018. Linking Synthetic Populations to Household Geolocations: A Demonstration in Namibia. *Data* 3(3), 30; DOI:10.3390/data3030030.

Table S1.1. Data sources for simulated population in Khomas, Namibia

Short name	Long name	Source, original unit	Output unit
Population			
dhs_hh	Individual recode file summarized by household	2013 Demographic and Health Survey [A]	region
dhs_geo	Geo-displaced cluster coordinates	2013 Demographic and Health Survey [A]	coordinate (cluster)
census_housing, census_person	20% microdata census sample	2011 Namibia Statistics Agency [B]	constituency
census_report	Final census report	2011 Namibia Statistics Agency [C]	constituency
Used to generate new spatial data			
Imagery_2014	High resolution satellite imagery	2014-2016 Maxar (DigitalGlobe) Quickbird imagery, 30cm [D]	Coordinate (2016 household)
Imagery_2004	High resolution satellite imagery	2004-2013 Maxar (DigitialGlobe) SPOT imagery, 40cm [D]	Coordinate (2001, 2006, 2011 household)
census_ea	2011 Census EA & constituency boundaries	2011 Namibia Statistics Agency [E]	EA, constituency
Auxiliary data			
ccilc_dst011_2012	Dist to land-cover: Cultivated terrestrial lands	2008-2012 GlobCover, 300m [F]	100m
ccilc_dst040_2012	Dist to land-cover: Woody / Trees	2008-2012 GlobCover, 300m [F]	100m
ccilc_dst130_2012	Dist to land-cover: Shrubs	2008-2012 GlobCover, 300m [F]	100m
ccilc_dst140_2012	Dist to land-cover: Herbaceous	2008-2012 GlobCover, 300m [F]	100m
ccilc_dst150_2012	Dist to land-cover: Other vegetation	2008-2012 GlobCover, 300m [F]	100m
ccilc_dst190_2012	Dist to land-cover: Urban	2008-2012 GlobCover, 300m [F]	100m
ccilc_dst200_2012	Dist to land-cover: Bare	2008-2012 GlobCover, 300m [F]	100m
cciwat_dst	Dist to water bodies	2000 OSM [G]	100m
dmsp_2011	Night-time lights intensity	2012 Suomi VIIRS, 500m [H]	100m
gpw4coast_dst	Dist to coastline	GPWv4, 1km [I]	100m
osmint_dst	Dist to road intersections	2000 OSM [G]	100m
osmriv_dst	Dist to major water ways	2000 OSM [G]	100m
slope	Slope	2000 HydroSHEDS, 100m [J]	100m
topo	Elevation	2000 HydroSHEDS, 100m [J]	100m
tt50k_2000	Travel time to populated places	2000 JRC-EC [K]	100m
urbpx_prp_1_2012	Proportion of urban pixels within 1 cell radius	2009 Modis [L,M]; Global Human Settlement City Model, 1km [N]	100m
hfacilities_dst	Dist to health centre or hospital	2001 UN-OCHA [O]	100m
schools_dst	Dist to primary/secondary school	2001 UN-OCHA [P]	100m
npp_2012	Annual net primary productivity	2010 MODIS, 1km [Q]	100m

A. ICF International. 2020. Available datasets. https://dhsprogram.com/data/available-datasets.cfm
B. NSA. 2013. Namibia 2011 Population and Housing Census version 1.0. https://nsa.org.na/microdata1/index.php/catalog/19
C. NSA. 2011. Namibia Population and Housing Census 2011 main report. http://www.nsa.org.na/files/downloads/Namibia 2011 Population and Housing Census Main Report.pdf
D. Maxar. 2019. Satellite Imagery. www.digitalglobe.com/products/satellite-imagery
E. NSA. 2011. 2011 Census EA boundaries. https://nsa.org.na/page/gis-data-requests/
F. European Space Agency. 2012. GlobCover. www.esa-landcover-cci.org/?q=node/158
G. OpenStreetMap contributors. 2000. OpenStreetMap base data. www.openstreetmap.org
H. NOAA. 2012. VIIRS nighttime lights. https://maps.ngdc.noaa.gov/viewers/VIIRS_DNB_nighttime_imagery/index.html
I. CIESIN. 2018. Gridded Population of the World, Version 4.11 (GPWv4.11). DOI:10.7927/H4F47M65
J. Lehner B, Verdin K, Jarvis A. 2006. HydroSHEDS technical documentation. www.worldwildlife.org/freshwater/pubs/HydroSHEDS_TechDoc_v10.pdf
K. Nelson A. 2008. Travel time to major cities: A global map of accessibility. https://forobs.jrc.ec.europa.eu/products/gam/
L. Schneider A, Friedl MA, Potere D. 2009. A new map of global urban extent from MODIS satellite data. Environ Res Lett;4:1–11. DOI: 10.2307/2346830.
M. Schneider A, Friedl MA, Potere D. 2010. Mapping global urban areas using MODIS 500-m data: New methods and datasets based on "urban ecoregions." Remote Sens Environ;114:1733–46. DOI:10.1016/j.rse.2010.03.003.
N. European Commission. 2017. Global human settlement city model (GHS-SMOD). http://ghsl.jrc.ec.europa.eu/faq.php
O. UN-OCHA-ROSA. 2001. Namibia health facilities. HDX. https://data.humdata.org/organization/ocha-rosa
P. UN-OCHA-ROSA. 2001. Namibia education facilities. HDX. https://data.humdata.org/organization/ocha-rosa
Q. Steven W. R, Ramakrishna R. N, Faith Ann H, et al. 2004. A continuous satellite-derived measure of global terrestrial primary production. Bioscience;54(6):547–60. DOI:10.1641/0006-3568(2004)054[0547:ACSMOG]2.0.CO;2

(3) In step 3, we calculated the main type of household in each 2013 DHS primary sampling unit (PSU) (550 nationally) based on k-means groups defined in Khomas (step 1), and joined the 2km averaged auxiliary data values (step 2) to each PSU point. The distribution of PSU main household type across Namibia was: 185 (34%) urban non-poor, 82 (15%) urban poor, 249 (45%) rural poor, and 34 (6%) rural non-poor. We used these 550 PSU household types as training data, and the average 2km covariate values in a Random Forest machine classification model to predict a probability surface for each household type in each 100x100m cell in Namibia. This model performed well for urban non-poor households (14.6% misclassification) and rural poor households (7.6% misclassification), though classification error was high in areas comprised of mostly urban poor households (58.5% misclassification) and rural non-poor households (76.5% misclassification) (Table S1.2). Errors within urban areas were expected because auxiliary data 2km buffers can mask disparities between neighbourhoods. Although expected, poor performance of the model for urban poor households was problematic and addressed in the next step. Misclassification of rural non-poor households was also not surprising given the small size of this population, though this problem was ignored because non-poor rural households comprised a very small portion of the population in Khomas (<1%).

Table S1.2. Random Forest confusion matrix for average household type in 550 DHS clusters in the Khomas, Namibia simulation

	**Type 1 –** Urban non-poor	**Type 2 –** Urban poor	**Type 3 –** Rural poor	**Type 4 –** Rural non-poor	**Classification Error**
**Type 1 –** Urban non-poor	158	23	3	1	0.146
**Type 2 –** Urban poor	40	34	7	1	0.585
**Type 3 –** Rural poor	8	3	230	8	0.076
**Type 4 –** Rural non-poor	4	0	22	8	0.765

(4) To improve the accuracy of the urban household probability layers in Khomas, we created an urban poor/non-poor weights layer by manually assigning each census EA with the portion of population that appeared to be located in a slum or informal settlement in 2016 based on visual inspection of 30cm Quickbird satellite imagery. Before beginning this process, we split large EAs at the periphery of Windhoek to create new EAs for areas that had undergone urban expansion since the 2011 census boundaries were drawn (total of 922 EAs). Rural EAs had a null probability in this step. The poor/non-poor weights layers were multiplied by the predicted household probability surfaces (step 3) to produce final 100x100m household probability surfaces (Figure S1.2).

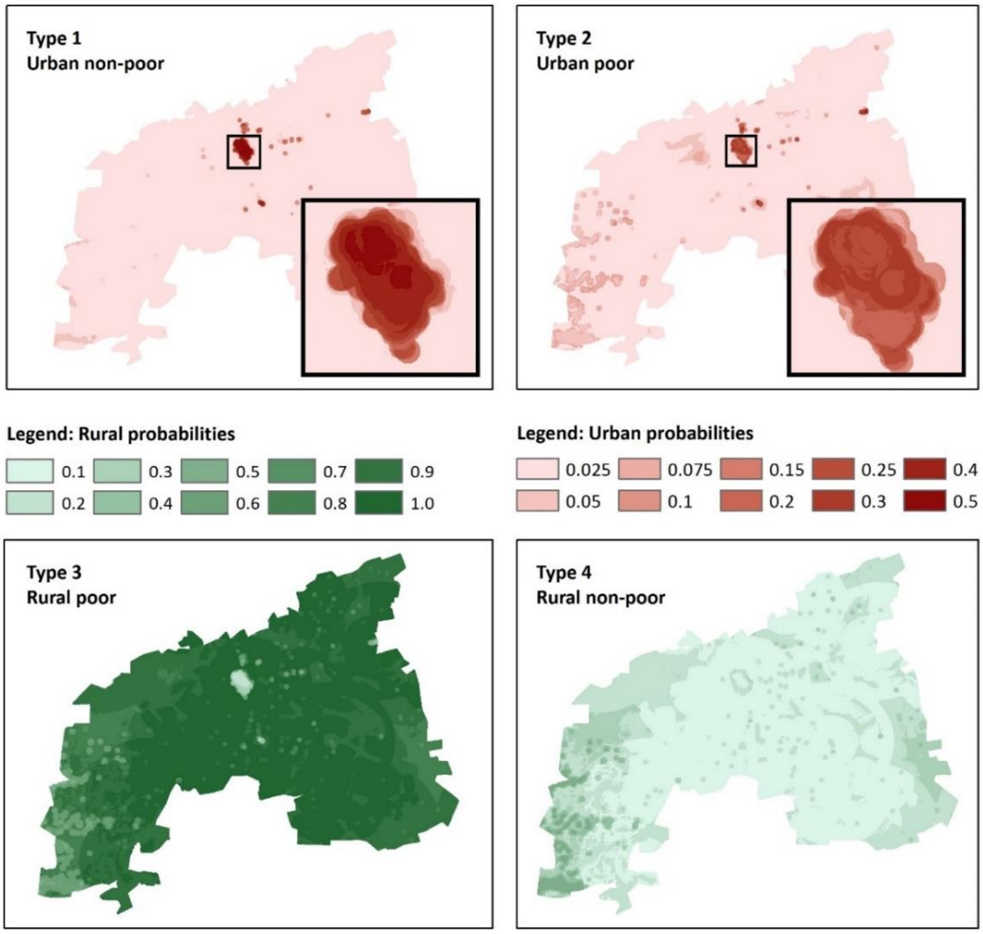

Figure S1.2. Household type probability surfaces (steps 1-4) in Khomas, Namibia population simulation

(5) In step 5, we manually digitized building locations across Khomas using 2014-2016 high-resolution (30cm) Quickbird imagery in ArcGIS 10. Subjective judgement was required; for example, deciding not to digitize some buildings on main streets in densely populated areas where shops and offices seemed likely. In areas of dense settlement, some points were duplicated to represent more than one household in the same building. A total of 97,667 household points were digitized in 2016. As a benchmark, we exported points to Google Earth and used 2011 Maxar and SPOT (40cm) imagery to identify buildings that were missing in 2011, and ensured that the reduced number of points matched constituency household counts in the 2011 census (Table S1.1, C).

(6) In step 6, we simulated a population of realistic households in Khomas using iterative proportional fitting (IPF) with combinatorial optimisation in the R *simPop* package [2] (Table S1.3). IPF starts by defining a basic household structure to ensure the synthetic population is realistic. We defined household structure with household size, urban/rural residence, and age and sex of household head at the household-level; and age, sex, and relationship (to head) at the individual-level. Inputs to the model were the 2011 Census 20% microdata sample, as well as urban and rural household sizes, and constituency population by age, sex, and relationship based on the 2011 census report (Table S1.1, C). The IPF model selects random samples of records from the microdata with replacement until each of the household structure targets per constituency are met.

[2] Templ M, Meindl B, Kowarik A, et al. 2017. Simulation of synthetic complex data: The R package simPop. J Stat Softw;79(10):1–38. www.jstatsoft.org/v79/i10/

Table S1.3. Iterative proportional fitting of household structure in Khomas, Namibia simulation by constituency

	Tobias Hainyeko	Katutura Central	Katutura East	Khomasdal North	Soweto	Samora Machel	Windhoek East	Windhoek Rural	Windhoek West	Moses Garoëb
N	60553	30868	24078	60465	19570	80036	27309	30028	62588	62807
**HH Size**										
Average	5.49	5.49	5.49	5.49	5.49	5.49	5.49	5.49	5.49	5.49
**Residence**										
Urban	100%	100%	100%	100%	100%	100%	100%	26%	100%	100%
Rural	0%	0%	0%	0%	0%	0%	0%	74%	0%	0%
**Relationship**										
Head	27%	21%	20%	24%	22%	26%	34%	30%	28%	30%
Spouse	10%	6%	5%	9%	6%	8%	18%	13%	13%	9%
Child	26%	27%	27%	31%	25%	27%	28%	28%	29%	23%
Grandchild	4%	8%	12%	4%	10%	6%	1%	7%	2%	5%
Extended	29%	31%	29%	26%	31%	28%	12%	14%	20%	29%
Other	5%	8%	7%	6%	5%	5%	8%	7%	8%	5%
**Sex**										
Female	45%	55%	56%	53%	53%	52%	51%	46%	53%	47%
Male	55%	45%	44%	47%	47%	48%	49%	54%	47%	53%
**Age**										
<1	4%	2%	3%	3%	2%	3%	2%	3%	2%	4%
1 - 4	9%	8%	9%	8%	7%	9%	7%	9%	7%	9%
5 - 9	9%	10%	10%	9%	9%	8%	6%	10%	7%	8%
10 - 14	8%	10%	10%	10%	9%	9%	6%	10%	8%	6%
15 - 19	8%	11%	11%	11%	11%	10%	8%	9%	11%	7%
20 - 24	15%	12%	13%	14%	17%	15%	8%	9%	15%	14%
25 - 29	14%	12%	10%	10%	12%	14%	9%	8%	10%	15%
30 - 34	11%	10%	8%	9%	9%	11%	9%	7%	9%	13%
35 - 39	9%	7%	7%	8%	6%	7%	9%	7%	7%	11%
40 - 44	6%	5%	5%	6%	4%	5%	9%	7%	6%	6%
45 - 49	4%	4%	4%	5%	3%	4%	6%	5%	5%	4%
50 - 54	2%	3%	3%	3%	4%	2%	6%	5%	4%	2%
55 - 59	1%	2%	2%	2%	3%	2%	5%	3%	3%	1%
60 - 64	1%	1%	2%	1%	1%	1%	3%	3%	2%	1%
65 - 74	0%	1%	2%	1%	1%	1%	5%	4%	2%	0%
75+	0%	1%	1%	1%	0%	0%	2%	2%	1%	0%

Next, using the R *simPop* package, we added household and individual characteristics present in the 20% microdata census dataset (toilet, water, structure, space, fuel, education) to the simulated dataset using a multinomial logistic regression technique and conditional annealing (Table S1.4**Error! Reference source not found.**). This treated age, sex, relationship, household size, and urban/rural residence as predictors, and each of the household characteristic as a conditional outcome.

We confirmed that there were not major differences between the distributions of characteristics in the 20% microdata and simulated dataset (all differences were less than +/- 0.002). Confident that the simulated household and individual characteristics were realistic, we calculated the most likely household type for each household based on variable factor weights created in the k-means analysis in step 1.

The 2011 census microdata sample was provided with a weight of approximately five for each observation to scale the 20% microdata sample to the total population in 2011. We calibrated the simulation to create an extra 20% of households to ensure there were enough simulated households to assign to 2016 point locations; left over simulated households were discarded in step 7. This resulted in 122,079 simulated households in Khomas before assignment to point locations.

Table S1.4. Multinomial logistic regression output of household characteristics in Khomas, Namibia simulation by constituency

	Tobias Hainyeko	Katutura Central	Katutura East	Khomasdal North	Soweto	Samora Machel	Windhoek East	Windhoek Rural	Windhoek West	Moses Garoëb
N (individuals)	60553	30868	24078	60465	19570	80036	27309	30028	62588	62807
**Water**										
Improved	100%	100%	100%	100%	100%	100%	100%	96%	100%	100%
Unimproved	0%	0%	0%	0%	0%	0%	0%	4%	0%	0%
**Toilet**										
Improved	25%	58%	67%	76%	69%	44%	97%	52%	94%	24%
Unimproved	75%	42%	33%	24%	31%	56%	3%	48%	6%	76%
**Floor**										
Durable	44%	97%	99%	88%	96%	72%	96%	80%	98%	44%
Non-durable	56%	3%	1%	12%	4%	28%	4%	20%	2%	56%
**Space**										
Adequate	81%	64%	64%	78%	74%	74%	96%	75%	93%	81%
Inadequate	19%	36%	36%	22%	26%	26%	4%	25%	7%	19%
**Fuel**										
Non-solid	87%	99%	97%	93%	99%	94%	100%	50%	100%	92%
Solid	13%	1%	3%	7%	1%	6%	0%	50%	0%	8%
**HH Head Education**										
No formal	24%	20%	21%	18%	16%	21%	14%	30%	14%	24%
Some primary	22%	20%	19%	19%	17%	18%	10%	24%	12%	20%
Primary	37%	38%	35%	32%	32%	36%	14%	28%	18%	38%
Secondary	15%	19%	20%	22%	26%	21%	33%	12%	32%	18%
Tertiary	2%	3%	5%	9%	8%	4%	29%	6%	24%	1%

(7) In step 7, we joined the re-weighted household type probabilities created in step 4 to the household latitude-longitude coordinates created in step 5. For each latitude-longitude coordinate created for 2016 household point locations, we randomly sampled a simulated household created in step 6 from the corresponding constituency and urban/rural strata based on the probabilities of household types at each coordinate. We repeated assignment of simulated households to coordinate point locations until all coordinates were assigned a simulated household, and then discarded the extra unassigned simulated households for a total of 97,667 simulated households located at realistic coordinate locations in Khomas for 2016.

(8) In step 8, we used the 2013 DHS records in Khomas (n=931 households) to develop multinomial models in R to simulate the same three individual and household outcomes as Thomson and colleagues (2018): household wealth quintile (five ordinal categories), woman's use of modern contraception (binary in women age 15 to 49), and child's receipt of 3rd DPT vaccination (binary in children under five) (Table S1.5). We used a multinomial model to calculate associations between each outcome and household-level covariates in the 2013 DHS dataset, and applied coefficients to the simulated dataset to predict wealth quintile, modern contraceptive use, and receipt of 3rd DTP vaccine for each household, woman 15 to 49, and child under five, respectively.

Table S1.5. Multinomial model coefficients and fit statistics for three outcomes in the 2013 DHS for Khomas, Namibia

Predictor	Household wealth quintile (ref=poorest)				Women 15-49 use of modern contraception	Child <5 DPT3 vaccination coverage
	poorer	middle	richer	richest		
Rural	0.479	0.773*	2.299***	2.061***	-0.227**	2.334***
HH Head						
15-29	(ref.)	(ref.)	(ref.)	(ref.)		
30-49	-11.595***	-11.222***	-11.581***	-10.890***		
50+	-9.957***	-9.171***	-8.901***	-7.715***		
HH Head Female	1.003***	0.778**	0.929**	0.333		
Age						
15 – 19					-1.290***	
20 – 24					-0.111**	
25 - 29					0.208***	
30 – 34					(ref.)	
35 – 39					0.030	
40 - 44					0.123**	
45 - 49					-0.023	
Child age 1 – 4						0.795***
Female						-0.188***
HH Head						
No education	(ref.)	(ref.)	(ref.)	(ref.)	(ref.)	(ref.)
Some primary	0.133	-0.133	0.121	0.166	0.562***	0.680***
Primary	1.459***	2.243***	2.401***	3.216***	-0.038	0.447***
Secondary	0.466	1.651***	2.675***	4.092***	0.023	0.258
Tertiary	4.844***	6.455***	7.491***	9.515***	-0.259***	0.667***
Water Unimproved	-1.262*	0.429	-106.655	-0.169	-0.023	11.129
Toilet Unimproved	-23.935***	-26.157***	-28.908***	-30.603***	-0.018	0.021
Space Inadequate	-0.771**	-1.652***	-0.292	-1.216***	0.028	0.293***
Floor Non-durable	-21.756***	-22.962***	-24.338***	-26.003***	0.297***	0.748***
Fuel Solid	-19.316***	-20.937***	-23.301***	-105.303***	-0.197**	-0.621***
Constant	77.205***	80.003***	82.729***	82.498***	0.446***	-0.250
AIC	30,400				27,470	6,344

Note: *p<0.1; **p<0.05; ***p<0.01

(9) To check the realism of this dataset, we compared the distribution of simulated household and individual outcomes (summarised by census enumeration areas - EAs) to households and individuals measured in the 2013 DHS (summarised by primary sampling units – PSUs) in Figure S1.3. The distribution of household characteristics appeared to be consistent between the simulated and DHS populations. However, individual characteristics were less consistent, and more heaped around the mean in the simulated dataset (Figure S1.3). This may have occurred because there were more observations per unit (EA vs PSU) in the simulated dataset, and more census units (922 EAs) compared to the 2013 DHS dataset (53 PSUs). Due to these inconsistencies, we only report household-level outcomes in the simulated dataset.

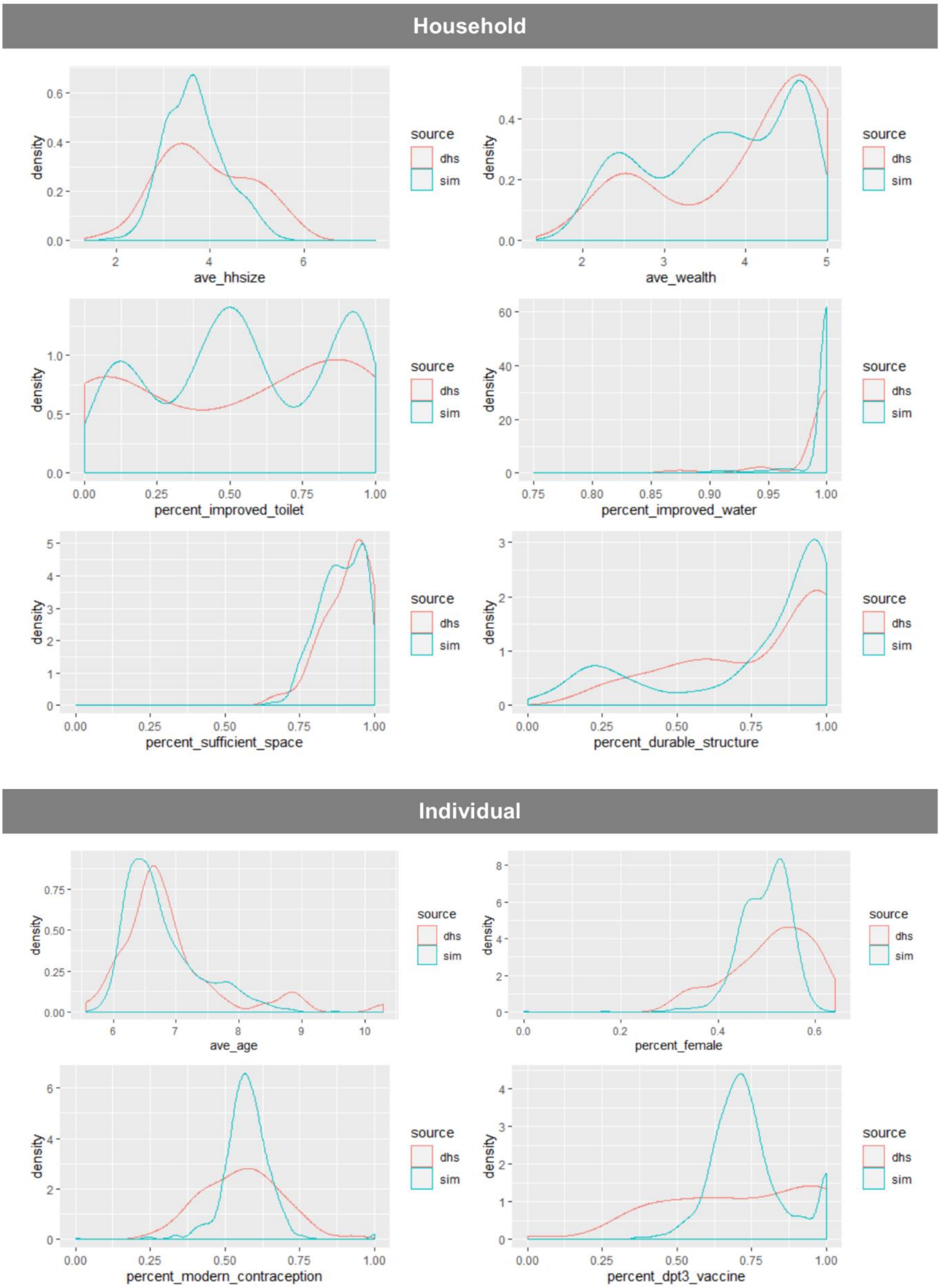

Figure S1.3. Comparison of household and individual outcomes by 2013 Namibia DHS cluster (n=53) and simulated population EA (n=922) in Khomas, Namibia
