# Supplementary material for: How accurate are WorldPop-Global-Unconstrained gridded population data at the cell-level?: A simulation analysis in urban Namibia
Source: PLoS One. 2022 Jul 21;17(7):e0271504. doi: 10.1371/journal.pone.0271504 (PMC9302737; doi:10.1371/journal.pone.0271504)
Supplement: Attachment — Submitted filename: cell_accuracy_response1.docx [file pone.0271504.s005.docx]

30 September 2021

Dear Dr. Vadrevu,

Thank you for this opportunity to revise and resubmit our manuscript titled “How accurate are WorldPop-Global-Unconstrained gridded population data at the cell-level?: A simulation analysis in urban Namibia”. We have responded point-by-point to editor and reviewer comments below in *italics*, and made corresponding revisions to the manuscript in track changes.

**Editor:**

When submitting your revision, we need you to address these additional requirements.

1. Please ensure that your manuscript meets PLOS ONE's style requirements, including those for file naming. The PLOS ONE style templates can be found at
   <https://journals.plos.org/plosone/s/file?id=wjVg/PLOSOne_formatting_sample_main_body.pdf> and <https://journals.plos.org/plosone/s/file?id=ba62/PLOSOne_formatting_sample_title_authors_affiliations.pdf>

*We have revisited and applied the PLOS ONE style templates to our manuscript. This included reformatting some headers and editing the references.*

2. Please note that in order to use the direct billing option the corresponding author must be affiliated with the chosen institute. Please either amend your manuscript to change the affiliation or corresponding author, or email us at [plosone@plos.org](mailto:plosone@plos.org) with a request to remove this option.

*Corresponding author Dr. Thomson has added her affiliation with the University of Twente.*

3. We note that Figures 1 & 3 in your submission contain [map/satellite] images which may be copyrighted. All PLOS content is published under the Creative Commons Attribution License (CC BY 4.0), which means that the manuscript, images, and Supporting Information files will be freely available online, and any third party is permitted to access, download, copy, distribute, and use these materials in any way, even commercially, with proper attribution. For these reasons, we cannot publish previously copyrighted maps or satellite images created using proprietary data, such as Google software (Google Maps, Street View, and Earth). For more information, see our copyright guidelines: <http://journals.plos.org/plosone/s/licenses-and-copyright>.
    
   We require you to either (1) present written permission from the copyright holder to publish these figures specifically under the CC BY 4.0 license, or (2) remove the figures from your submission:

*The maps in figs 1 and 3 were generated with publicly available, open datasets – we have added source information to each figure sub-title. We also dropped Supplement Fig 4 which was produced with proprietary satellite imagery.*

**Reviewer #1**

I find the topic interesting and the authors show to be experts in the topic. However, I think that the paper is too technical for most potential readers. I provide some simple comments hoping they can help in this regard.

4. The abstract is extremely and unnecessarily long.

*The abstract was formerly 300 words, meeting the PLOS ONE requirement. We have shorted it slightly, while also editing it for a more general audience.*

5. I find the intro too technical. I recommend leaving technical thing for other sections and devoting more in the intro to tell the reader i) why is the topic relevant for policy debates, ii) what are the main contribution of the current paper?

*Text about why it is important to evaluate the accuracy of gridded population datasets in LMICs has been moved earlier in the introduction. We also shortened the paragraph that summarises gridded population datasets in LMICs, and added several sentence at the end of the introduction to clarify the contribution of this paper, and its purpose: “This is among the first assessments of cell-level accuracy of a gridded population dataset in a LMIC setting. While the methods and approach outlined here (developing a realistic synthetic population, and from this deriving several realistic versions of census data) were applied to just one gridded population dataset, the approach could be applied to other gridded population datasets thereby enabling much needed assessments and comparisons of cell-level accuracy across gridded population data products used for development monitoring and decision-making.”*

6. I like the idea of a short and synthetic paper. However, even in a paper of this style, I think framing the paper in the relevant literature is essential. There are several papers on urbanisation, urban density, suburbanisation, etc. worldwide that the paper should cite and relate to. I recommend recent papers in the Journal of Economic Geography, Journal of Development Studies and in the Journal of Urban Economics.

*We cite several additional articles from the urban science literature in the introduction to underscore the importance of accurate, timely fine-scale population data which reflects the heterogeneity and dynamism of LMIC cities to monitor and address urban economic development.*

7. Could the text be easier to read, leaving some technicalities for an appendix?

*We have already moved most of the technical details to Supplement 1, though, we have added numbered lists to the methods to help delineate the different methodological steps that are summarised in this section.*

8. Sorry but I find really puzzling the use of “simulated” and “true” in the same sentences over and over in the paper to refer to the same numbers. How can be, at the same time, “simulate” and “true”?!

*The alternative description of our approach is that we simulated a realistic* synthetic *population for a real-world location. Describing our synthetic population as a “true” population (quotes always included and emphasised) made sense to some non-technical colleagues while we performed the work. However, perhaps our attempts to write for a general audience here, were misjudged. We have replaced* “true” *with the term* synthetic *or* “true” synthetic *throughout the paper. We hope this is clearer.*

9. How could we extrapolate the findings for Namibia to other wold regions?

*We have to be careful not to draw global conclusions from this simulated study in one city. However, as Reviewer 2 alluded to, the approach we used to evaluate one gridded population dataset in this one setting is replicable and scalable to other settings and datasets. We added the following sentence to the discussion: “Given inaccuracies of WorldPop-Global-Unconstrained, and methodological similarities with other gridded population estimates, this analysis both raises flags about cell-level inaccuracies across all gridded population products in high-density urban areas, a problem being underscored elsewhere [94], and offers a methodological approach that can be replicated in other settings to evaluate the accuracy of any gridded population dataset at the cell-level.”*

10. Finally, I miss a connection with applied research. For user of data sources like Gridded Population of the World, what does all mean? What are the implications? Alternatives? Etc. I think the authors should make discuss all this, leaving technicalities aside, in the conclusions

*We previously speculated about how these findings relate to other gridded population datasets that are available, but we have rearranged the discussion to emphasise the sources of potential inaccuracies with other gridded population datasets, and solutions that might improve their accuracy.*

*Because the conclusion should focus on key messages derived from our analysis (not speculation), we did not make changes to this section. The key take-away from this paper is: grid-cell level analysis is important to evaluate, especially in LMIC gridded population datasets where gridded population data is already being used to in development initiatives, and we demonstrate how this can be done with realistic simulated data.*

**Reviewer #2:**

11. As the authors point out, the paper presents a method of evaluating the cell-level accuracy of 32 simulated 100x100m WorldPop-Global-Unconstrained gridded population datasets which reflect realistic scenarios of census (1) outdatedness, (2) inaccuracy, and (3) aggregation in an urban LMIC setting. This topic is very interesting and timely, but the purpose of the article should be described more clearly.

*This comment is similar to comment 5 by Reviewer 1, so please see above.*

12. A thorough overview of the literature is included in Introduction, and the quoted items exhaust the proposed topic. In line 123 authors state that they evaluate 32 simulated 100x100m WorldPop-Global-Unconstrained gridded population datasets. The authors should explain why they chose the 32 grid. What was the reason for choosing such a set of gridded population datasets?

*We have clarified that “This paper describes how we evaluated the cell-level accuracy of 32 simulated 100x100m WorldPop-Global-Unconstrained gridded population datasets which reflect realistic levels of census (1) outdatedness (0-, 5-, 10-, and 15-years outdated), (2) inaccuracy (none, low, middle, and high missing population counts), and (3) two administrative-level aggregations of the population in an urban LMIC setting.” This means that 32 is the product of 4 x 4 x 2 scenarios.*

13. The section Methods is well presented and illustrated with figures. Yet, it should be explained why the Root Mean Square Error (RMSE) was selected to evaluate the gridded population dataset. The literature provides ample evidence on the effectiveness and usefulness of the Mean Absolute Error (MAE).

*We calculated both RMSE and MAE in an early version of this work (see DRT’s PhD thesis, available here:* [*http://eprints.soton.ac.uk/id/eprint/450772*](http://eprints.soton.ac.uk/id/eprint/450772)*). RMSE and MAE statistics in this analysis are nearly perfectly correlated, so we chose to present only RMSE because (a) there are already multiple input data scales, output scales, and scenarios presented, and (2) because RMSE penalises large errors, and therefore is slightly more conservative than MAE. Since we have calculated MAE, let us know if you and/or the editor would like us to add 4 tables to Supplement 4 with: (1) Area-adjusted MAE for EA-level input, (2) Area-adjusted MAE for Constituency-level input, (3) Population-adjusted MAE for EA-level input, and (4) Population-adjusted MAE for Constituency-level input. We hesitate to add these additional tables now because they seem redundant, and do not add information to an already complex analysis.*

14. The quality of figures and charts is quite unsatisfactory, and they need to be presented in adequate resolution.

*All of the submitted figures are 300+ dpi, but most were smaller than a full page. The resolution quality looks poor in the auto-generated PDF circulated to reviewers because each figure is enlarged to a full page (beyond the original resolution). The images should be clear when printed at the original resolution or smaller.*

15. In Figure 1, the black background interferes with map reading. What do white boundaries on the right of the map mean?

*We improved Figure 1 by adding a legend for the white lines (constituency boundaries). The black colour of the Windhoek map corresponds with the fill colour of Khomas region in the overall Namibia map.*

16. The Discussion section is presented in a clear way. Will the studies be continued?

*A motivation for this work was to raise the issue of cell-level accuracy as an ethical priority among gridded population dataset* producers *so that they might evaluate and address methodological issues in the data production pipeline before datasets are in the hands of data users (and able to influence decision-makers). The co-authors have been successful in motivating some others in our data community to tackle this issue. In the discussion, we now mention and cite one such initiative, which is currently being expanded to more than a dozen additional cities globally. That text is: “...this analysis... raises flags about cell-level inaccuracies across all gridded population products in high-density urban areas, a problem being underscored elsewhere [94]...”.*

17. Is the proposed method universal? Can it be used for other research areas?

*Yes, the methodological approach can be applied universally to other contexts and gridded population datasets. We are now clearer about this point throughout the paper.*

18. The Conclusions section reinstates the main findings in an adequate way. The article meets high scientific quality standards and fits the scope of PLOS ONE. It contributes to the existing knowledge, presenting the topic in an interesting and up-to-date way.

*Many thanks to both reviewers for their thoughtful reviews. The comments and suggestions have helped to improve the manuscript.*

Please do not hesitate to contact me with any questions or concerns.

Most sincerely,

Dana R. Thomson (with Douglas R. Leasure, Tomas Bird, Nikos Tzavidis, and Andrew J. Tatem)
