# Supplementary material for: How accurate are WorldPop-Global-Unconstrained gridded population data at the cell-level?: A simulation analysis in urban Namibia
Source: PLoS One. 2022 Jul 21;17(7):e0271504. doi: 10.1371/journal.pone.0271504 (PMC9302737; doi:10.1371/journal.pone.0271504)
Supplement: Attachment — Submitted filename: cell_accuracy_response2.docx [file pone.0271504.s006.docx]

31 January 2022

Dear Dr. Vadrevu,

Thank you for this opportunity to provide minor revisions to our manuscript, “How accurate are WorldPop-Global-Unconstrained gridded population data at the cell-level?: A simulation analysis in urban Namibia”. We have responded to comments below in *italics*, and made corresponding revisions to the manuscript in track changes.

**Reviewer #1**

1. Authors have not made all data underlying the findings in their manuscript fully available.

*It is unclear why the reviewer believes that the underlying findings are not fully available with our manuscript. All of the datasets that we used to simulate populations are publicly available and linked in the cited publication by Thomson et al. 2018 and in Table 3. The simulated outdated censuses are based on actual historical satellite imagery, which is publicly available and cited. Our parameters to define inaccurate censuses are based on a systematic literature search, which is described and cited. Finally, our simulated “true” population and all 32 versions of our simulated censuses are provided in Supplement 2. If we have missed any datasets, please let us know which ones and we will make them available or provide the corresponding links.*

2. I still find the paper very technical, but I understand that this is the contribution. My concern is that, given its focus and style, the reach of the paper will be limited (see my next comment). In this line, the new intro helps. The discussion had not changed much and could try to be broader in scope.

Related to the above, the literature continuous to be deficient. Most references are technical. For applied “users” of gridded data (rather than researchers “creating” or “adjusting” the data), one wants to relate to applied work using this data to study several outcomes that you mention in the intro (i.e., development, environmental outcomes, etc.). Aside some papers about vaccination and health outcomes, there are hardly any reference to this type of papers. Think that many of your potential readers will be authors in journal like the JouEcoGeo JouUrbEco, JourDevStud, etc. I recommended trying to relate to recent work in these journals. I see no reference.

*We appreciate the push to keep data users in mind because we, ultimately, hope this paper can impact how gridded population modellers measure and report accuracy, and thus improve the accuracy and usability of gridded datasets for users.*

*However, the stated focus of this paper is on a* ***creative approach to measure fine-scale accuracy*** *of a gridded population dataset (see last paragraph of introduction). Broadly, the implications are the same for all indicators and sectors, so we have added the following sentence to the first paragraph of the discussion, “In practical terms, this means that urban development indicators calculated with a WorldPop-Global-Unconstrained dataset at fine scale (e.g., neighbourhood) would likely be incorrect, and could lead to confusing results. For example, an underestimate of the number of people living in a neighbourhood could both make vaccination coverage rates as well as disease infection rates appear incorrectly high in that neighbourhood.”*

*However, we do not intend to broaden the scope of the discussion further to specific use cases of one or more gridded population datasets because the results do not support this.*

*Note that in our last revision, we added discussion of how our findings about the WorldPop-Global-Unconstrained model might translate to other gridded population models if assessed for accuracy in the same way. We also cited additional urban development journals, including Environ Urban, in the opening paragraph while listing potential uses cases for fine-scale gridded population estimates.*

*We hope these explanations and edits are acceptable to the reviewer.*

3. Try to shorten (or break) sentences were possible.

*We split longer sentences in several places throughout the paper (e.g. lines 94, 104, 147, 183).*

**Reviewer #2:** “The authors adequately addressed the comments of reviewers. I believe that this manuscript is now acceptable for publication.”

We thank both reviewers for their time and constructive feedback which has helped to strengthen the paper. Please do not hesitate to contact us with any questions or concerns.

Most sincerely,

Dana R. Thomson (with Douglas R. Leasure, Tomas Bird, Nikos Tzavidis, and Andrew J. Tatem)
