# Supplementary material for: How accurate are WorldPop-Global-Unconstrained gridded population data at the cell-level?: A simulation analysis in urban Namibia
Source: PLoS One. 2022 Jul 21;17(7):e0271504. doi: 10.1371/journal.pone.0271504 (PMC9302737; doi:10.1371/journal.pone.0271504)
Supplement: Attachment — Submitted filename: cell_accuracy_response3.docx [file pone.0271504.s007.docx]

2 July 2022

Dear Dr. Vadrevu,

Thank you for this opportunity to provide minor revisions to our manuscript, “How accurate are WorldPop-Global-Unconstrained gridded population data at the cell-level?: A simulation analysis in urban Namibia”. Please find our responses below in *italics* and track changes in the manuscript.

**Editor:**

1. Please revise manuscript to reflect application potential of the topic with relevant references.

*We have added the following paragraph to the discussion:*

*This analysis reinforces findings of other studies which find that currently available gridded population products tend to underestimate populations in urban areas [94–96], especially in higher-density poorer neighbourhoods [97]. For example, Tuholske and colleagues (2021) compared five gridded population products to estimate the proportion of population affected by natural disasters (SDG 11.5) in three regions where disasters had occurred, and found that 1x1 km population estimates varied widely among data products, and reflected anywhere from 20% to 80% of the total UN estimated population in each region. Furthermore, they found that WorldPop-Global-Unconstrained generally performed better than un-modelled products (e.g., GPW), but not as well as products that constrained estimates to settled cells (e.g., GHS-POP) [94]. In a separate comparison of nine gridded population estimates in Kenyan and Nigerian slum populations (SDG 11.1) where field counts were available for reference, the estimated population in each slum varied widely and WorldPop-Global-Unconstrained estimates reflected just 11% of the overall slum population while the best performing data product (HRSL) estimated just 34% of all slum dwellers [97]. A key take-away from gridded population comparison studies is that fine-scale accuracy across data products varies substantially depending on location, potentially leading to different conclusions and decisions (e.g., about the humanitarian need or health care burden) depending on the gridded population dataset used for analysis. Furthermore, these studies underscore the need to understand fine-scale accuracy across gridded population datasets and locations to inform improvements to the underlying modelling methods and inputs.*

2. Please review your reference list to ensure that it is complete and correct.

*We checked the references to ensure they are complete and correct, including URL links.*

**Review #1:**

3. I still find the paper very technical, but I understand that this is the contribution. My concern is that, given its focus and style, the reach of the paper will be limited (see my next comment). In this line, the new intro helps. The discussion had not changed much and could try to be broader in scope.

Related to the above, the literature continuous to be deficient. Most references are technical. For applied “users” of gridded data (rather than researchers “creating” or “adjusting” the data), one wants to relate to applied work using this data to study several outcomes that you mention in the intro (i.e., development, environmental outcomes, etc.). Aside some papers about vaccination and health outcomes, there are hardly any reference to this type of papers. Think that many of your potential readers will be authors in journal like the JouEcoGeo JouUrbEco, JourDevStud, etc. I recommended trying to relate to recent work in these journals. I see no reference.

*We did not find any studies that applied or evaluated gridded population datasets in the Journal of Economic Geography, Journal of Urban Ecology, and Journal of Development Studies. We still stand by our previous response to this comment – that we do not intent to discuss “specific use cases of one or more gridded population datasets because the results do not support this.” However, as detailed above, we did add a paragraph to the discussion about other gridded population accuracy assessments and comparison studies in the contexts of disaster response (SDG 11.5) and estimating slum populations (SDG 11.1).*

4. Try to shorten (or break) sentences were possible.

*We split a few additional sentences throughout the paper to improve readability (e.g., lines 52, 392) and made a few additional minor edits to improve readability.*

Please do not hesitate to contact us with any questions or concerns.

Most sincerely,

Dana R. Thomson (with Douglas R. Leasure, Tomas Bird, Nikos Tzavidis, and Andrew J. Tatem)
